# Bruton’s tyrosine kinase inhibitors in diffuse large B-cell lymphoma therapy: critical considerations and future innovations

**DOI:** 10.1186/s12967-026-07798-8

**Published:** 2026-03-23

**Authors:** Chuanyang Lu, Qiuni Chen, Yaodong Shen, Miaohong Chen, Yumeng Xie, Chunling Wang, Liang Yu

**Affiliations:** 1 Department of Hematology, The Affiliated Huaian No.1 People’s Hospital of Nanjing Medical University, Huai’an, 223300 China; 2 Department of Hematology, The Huaian Clinical College of Xuzhou Medical University, Huai’an, 223300 China; 3 Northern Jiangsu Institute of Clinical Medicine, Nanjing Medical University, Huai’an, 223300 China; 4 Key Laboratory of Autoimmune Diseases of Huaian City, Huai’an, 223300 China; 5 Key Laboratory of Hematology of Nanjing Medical University, Nanjing, 210029 China

## To the editor

Diffuse large B-cell lymphoma (DLBCL) is the most common subtype of aggressive non-Hodgkin’s lymphoma (NHL) and also the most common lymphoma in adults. Under the current first-line standard immunochemotherapy regimen R-CHOP (rituximab, cyclophosphamide, doxorubicin, vincristine, and prednisone) treatment, most patients can be cured [1]. However, there are still 30 ~ 40% of patients who either respond poorly to this treatment or face the challenge of recurrence post-treatment. The prognosis for patients with relapsed or refractory (R/R) DLBCL is even worse, necessitating the urgent development of novel therapeutic targets or strategies to prolong their survival and enhance clinical outcomes. The B-cell receptor (BCR) signaling pathway plays a critical role in regulating all stages of B-cell development, contributing to the proliferation, differentiation, and survival of B cells. The present studies have shown that aberrant activation of this pathway is implicated in the pathogenesis of various B-cell malignancies, including DLBCL. Bruton’s tyrosine kinase (BTK) is a key component of the BCR signaling pathway, mediating the transmission of BCR signals into the nucleus and triggering downstream pathways, including the nuclear factor-κB (NF-κB) pathway and the MAPK/ERK pathway, etc [2]. Consequently, targeting BTK has emerged as a significant therapeutic approach for treating B-cell malignancies. However, the overall therapeutic efficacy of BTK inhibitors (BTKi) in DLBCL remains modest. This is partly due to the high genetic heterogeneity of DLBCL, which complicates the application of BTK, as different genetic molecular subtypes exhibit varying responses to BTKi [3]. Additionally, the emergence of primary and secondary resistance to BTKi has become increasingly prominent in clinical practice, limiting the application and development of BTKi in DLBCL. Currently, combining BTKi with other treatment strategies, including chemotherapy regimens, immunotherapy, and other targeted therapies, holds promise for overcoming resistance to BTKi [4]. This research letter provides a comprehensive landscape analysis of clinical trials assessing BTKi in combination therapies for DLBCL, with a particular focus on trial designs, combination therapy regimens, and emerging trends.

**Fig. 1:**
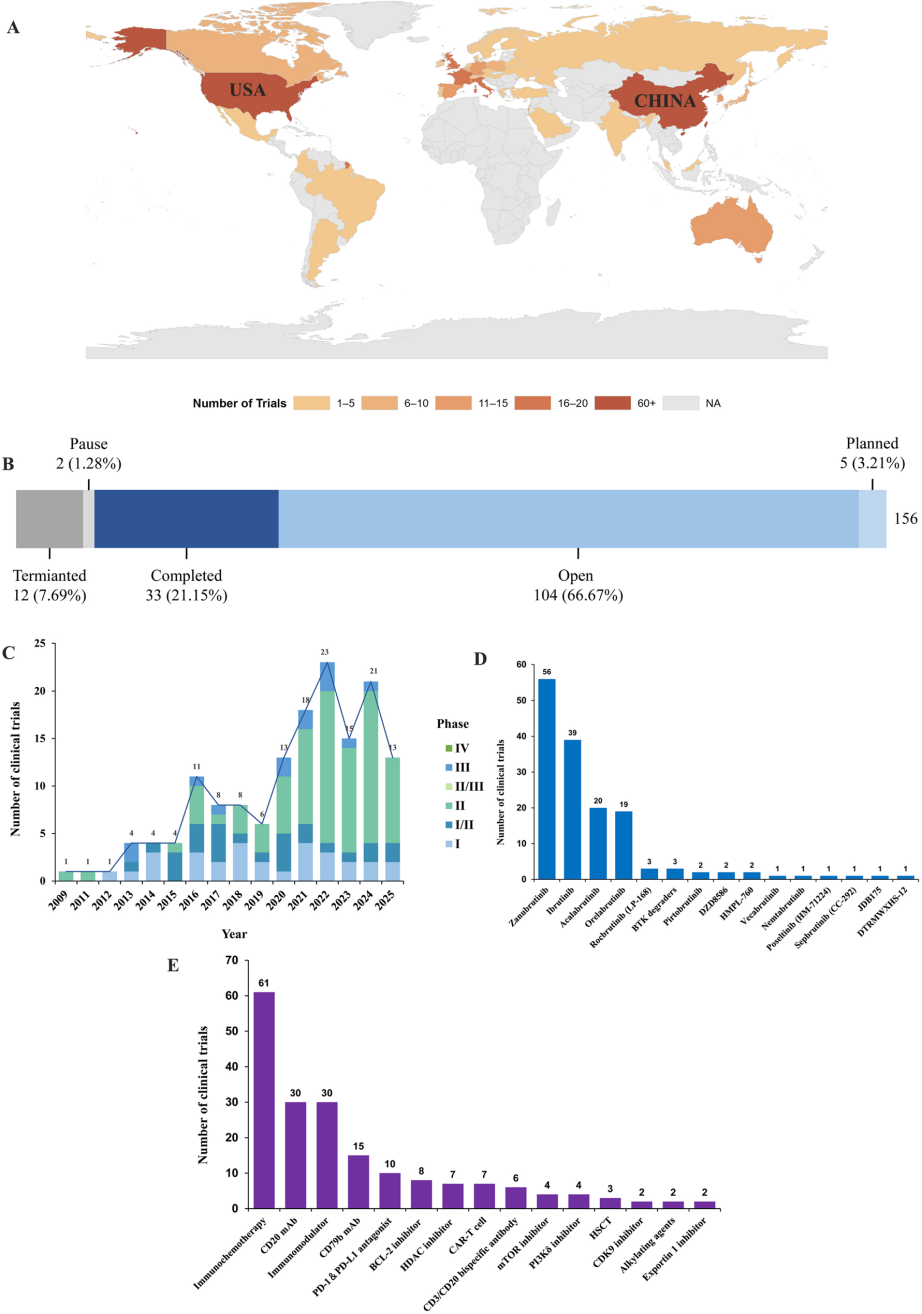
The global landscape of Bruton’s tyrosine kinase inhibitors (BTKi) in the treatment of diffuse large B-cell lymphoma (DLBCL): (**A**) Geographic distribution of countries where clinical trials on BTKi for DLBCL are registered. (**B**) Status of BTKi trials as of September 11, 2025. (**C**) The number and phases of clinical trials on BTKi for DLBCL from 2009 to 2025. (**D**) Ranking of specific BTKi based on their frequency of use in clinical trials. (**E**) Top 15 combination strategies employed in clinical trials of BTKi for DLBCL

A thorough search was conducted across the PharmCube and ClinicalTrials.gov databases, focusing on registered clinical trials targeting BTKi for the treatment of DLBCL. Among all registered clinical trials, China and the United States emerge as the top two countries in terms of trial registration (Fig. 1A). The majority of these trials are currently ongoing, with 21.15% having been completed to date (Fig. 1B). Since 2009, there has been a consistent year-on-year increase in the registration of clinical trials investigating BTKi for DLBCL. Most of these trials are in Phase I and II, while the number of trials in Phase III and IV remains relatively limited (Fig. 1C). The predominant type of BTKi utilized in clinical trials is irreversible covalent inhibitors. Among these, Zanubrutinib leads in terms of the number of registered trials, followed by Ibrutinib, Acalabrutinib, and Orelabrutinib. Reversible non-covalent BTKi constitute only a minor fraction of the trials. Additionally, a small number of clinical trials have been registered for BTK degraders (Fig. 1D). Combination therapy strategies centered around BTKi primarily involve immunochemotherapy regimens. Immunotherapy plays a pivotal role in these combination approaches. Furthermore, inhibitors targeting molecules that may contribute to BTKi resistance in B-cell malignancies, such as BCL-2, PI3K, mTOR, and nuclear exportin 1, form an integral part of the combination therapy landscape (Fig. 1E).

The incorporation of BTKi into combination therapies for DLBCL has demonstrated encouraging efficacy, particularly in high-risk and R/R settings. The underlying mechanisms involve the synergistic targeting of the BCR signaling pathway and enhanced immune activation when BTKi are combined with CAR-T therapy or bispecific antibodies. However, as previously noted, due to the molecular heterogeneity inherent in DLBCL, biomarker-driven strategies are imperative in subsequent clinical trials. These strategies should prioritize the evaluation of the efficacy and safety of BTKi across different genetic subtypes, with a particular focus on patients with non-germinal center B-cell (non-GCB) subtype or those harboring *MYD88/CD79B* co-mutations (MCD subtype). Moreover, the majority of currently registered clinical trials investigating BTKi in DLBCL are in relatively early phases. We eagerly anticipate the launch of subsequent larger-scale, multi-center clinical trials to further investigate the efficacy and safety of BTKi in DLBCL. Finally, compared to covalent BTKi, non-covalent BTKi exhibit efficacy in individuals with acquired BTK C481S mutations and have the potential to overcome resistance to irreversible BTKi. Consequently, non-covalent BTKi may emerge as a focal point of interest for researchers in future registered clinical trials. Meanwhile, developed dual-target inhibitors like DZD8586 (LYN/BTK inhibitor) more effectively block BCR signaling, showing good antitumor activity in various B-NHL subtypes including DLBCL. Additionally, BTK proteolysis targeting chimeras (PROTACs) using the ubiquitin-proteasome system provide higher selectivity, fewer side effects, and better efficacy while overcoming acquired BTK resistance [5]. These findings open new avenues for BTK inhibitor therapy in DLBCL.

BTKi combination therapies are reshaping DLBCL management, with emerging evidence supporting their role in high-risk and R/R disease. Future directions should focus on biomarker-driven trials, novel combinations, and real-world studies to validate long-term benefits. International collaboration and adaptive trial designs will be crucial to address unmet needs and ensure equitable access.

## Data Availability

The datasets used and analyzed during the study are available from the corresponding author on reasonable request.
